# Patient Safety Culture and Its Work-Related Predictors Among Hemodialysis Nurses in Jeddah, Saudi Arabia: A Multicentre Cross-Sectional Study Using the Hospital Survey on Patient Safety Culture

**DOI:** 10.7759/cureus.106021

**Published:** 2026-03-28

**Authors:** Nashwa Barnawi, Faridah Mohd Said

**Affiliations:** 1 Nursing, Lincoln University College, Petaling Jaya, MYS

**Keywords:** dialysis units, hemodialysis nurses, hsopsc, patient safety culture, safety climate, saudi arabia

## Abstract

Background

Patient safety culture is a critical determinant of healthcare quality, particularly in high-risk environments such as hemodialysis units, where complex procedures, advanced technology, and vulnerable patient populations increase the likelihood of adverse events. Despite global emphasis on patient safety, evidence regarding safety culture in hemodialysis settings within Saudi Arabia remains limited. This study aimed to assess patient safety culture and identify its work-related predictors among hemodialysis nurses in Jeddah, Saudi Arabia, using the Hospital Survey on Patient Safety Culture (HSOPSC).

Methods

A multicentre cross-sectional study was conducted among 400 nurses working in renal dialysis units across four hospitals in Jeddah. A convenience sampling technique was employed. Data were collected using a structured questionnaire comprising sociodemographic and work-related variables and the HSOPSC tool, which evaluates 12 dimensions of patient safety culture. Descriptive statistics were used to summarise participant characteristics and safety culture dimensions. Multivariable logistic regression analysis was performed to identify predictors of overall patient safety culture, with statistical significance set at p < 0.05.

Results

The overall patient safety culture score was moderate (mean = 3.29 ± 0.74). The highest-rated dimensions were management support for patient safety (mean = 3.37) and organisational learning/continuous improvement (mean = 3.36), while frequency of events reported scored the lowest (mean = 3.20). Most respondents rated patient safety as “very good” (n = 150, 37.5%) or “excellent” (n = 79, 19.8%), although 38.3% (n = 153) reported no safety events in the preceding 12 months. Significant predictors of higher patient safety culture included having 3-5 years of work experience (odds ratio (OR) = 2.32, p = 0.003), working less than 50 hours per week (OR = 1.64, p = 0.016), and reporting fewer or moderate numbers of safety events.

Conclusion

Patient safety culture among hemodialysis nurses in Jeddah is moderately positive but reveals critical gaps in event reporting and workload-related factors. Work experience, working hours, and organisational context significantly influence safety perceptions. Targeted interventions focusing on strengthening reporting systems, optimising staffing, and reinforcing organisational support are essential to enhance patient safety culture in hemodialysis settings.

## Introduction

Patient safety is a fundamental component of healthcare quality, reflecting a health system’s capacity to minimise preventable harm and optimise patient outcomes. Unsafe care continues to contribute significantly to global morbidity and mortality, emphasising the need for sustained system-level interventions and safety-focused organisational practices [1,2]. Contemporary perspectives increasingly recognise that patient safety is shaped not only by individual competence but also by organisational structures, processes, and culture, thereby necessitating a shift toward systems-based approaches to prevention and quality improvement [3,4].

Hemodialysis units represent a particularly high-risk healthcare environment due to the complexity of procedures, reliance on advanced medical technologies, and the fragile clinical status of patients with end-stage renal disease. The delivery of hemodialysis requires precise coordination of multiple clinical processes, including machine setup, vascular access management, anticoagulation therapy, and continuous monitoring of physiological parameters. Errors in these processes may result in serious complications such as intradialytic hypotension, bloodstream infections, or treatment failure [5,6]. Furthermore, the frequent use of invasive devices, including catheters and needles, significantly increases the risk of infection, underscoring the importance of stringent infection prevention and control practices [7]. These risks are further exacerbated by the high patient turnover and time-sensitive nature of dialysis care, which can increase workload pressures and the likelihood of human error [8].

Within this context, patient safety culture has emerged as a critical determinant of healthcare quality. Patient safety culture refers to the shared values, beliefs, and norms within an organisation that influence healthcare professionals’ attitudes and behaviours toward safety practices. A strong safety culture is characterised by effective teamwork, open communication, continuous organisational learning, and a non-punitive approach to error reporting [9]. The assessment of patient safety culture has been facilitated by validated instruments such as the Hospital Survey on Patient Safety Culture (HSOPSC), developed by the Agency for Healthcare Research and Quality (AHRQ), which evaluates multiple dimensions, including teamwork, communication openness, feedback mechanisms, and management support [10]. These dimensions provide a structured framework for identifying strengths and areas requiring improvement in healthcare organisations.

A growing body of literature highlights the role of structured interventions in enhancing patient safety within hemodialysis settings. Safety checklists, in particular, have gained prominence as an effective strategy for standardising clinical processes and reducing variability in care delivery. Evidence suggests that checklist implementation improves adherence to safety protocols, enhances communication among healthcare teams, and reduces adverse events across clinical settings [11-13]. In hemodialysis contexts, structured safety checklists have been associated with improved clinical outcomes, including reductions in vascular access complications and treatment-related errors [14-16].

Despite these advancements, significant challenges persist in fostering a robust patient safety culture in hemodialysis units. Underreporting of adverse events remains a critical concern, often influenced by fear of blame and punitive organisational responses. Moreover, work-related factors such as staffing shortages, extended working hours, and variability in institutional support can adversely affect nurses’ safety perceptions and practices [9]. These issues highlight the importance of examining not only the level of patient safety culture but also the underlying work-related determinants that influence it. Understanding these predictors is essential for designing targeted interventions that address both systemic and human factors contributing to patient safety.

In Saudi Arabia, research on patient safety culture has predominantly focused on general hospital settings, with limited attention to specialised areas such as hemodialysis. Given the increasing burden of chronic kidney disease and the growing demand for dialysis services in the country [17,18], there is a pressing need to evaluate safety culture within this high-risk clinical context. Furthermore, identifying key work-related predictors, such as workload, years of experience, and institutional differences, can provide actionable insights for healthcare leaders and policymakers seeking to improve safety outcomes and healthcare quality.

Therefore, this study aims to assess patient safety culture among hemodialysis nurses in Jeddah, Saudi Arabia, using the HSOPSC instrument and to identify the work-related factors that predict safety perceptions. By addressing existing gaps in the literature, this study contributes to the growing body of evidence required to strengthen patient safety strategies in hemodialysis units and similar high-risk healthcare environments.

## Materials and methods

We conducted a multicentre cross-sectional study among hemodialysis nurses at four hospitals in Jeddah, Saudi Arabia. Data were collected over one month using a structured questionnaire. Ethical approval was obtained from the Institutional Review Board of the Ministry of Health, Kingdom of Saudi Arabia (IRB log number: A02288) before the commencement of the study. All participants provided informed consent, and confidentiality and anonymity were strictly maintained throughout the study.

Study design and setting

This study employed a multicentre cross-sectional design to assess patient safety culture among nurses working in hemodialysis units in Jeddah, Saudi Arabia. Cross-sectional designs are widely used in healthcare research to evaluate perceptions and practices at a single point in time. They are appropriate for examining associations between variables in clinical settings [19]. The study was conducted across four hospitals (King Abdulaziz Hospital, Al Thaghr Hospital, East Jeddah General Hospital, and Adams Hospital) that provide renal dialysis services and represent diverse institutional contexts within the region.

Study population and sampling

The study population consisted of registered nurses and head nurses working in the selected hemodialysis units. A total of 400 participants were recruited for the study, with the sample size determined using an online sample size calculator based on the maximum possible population of eligible nurses in the selected units. Convenience sampling was employed to recruit participants who were available and willing to participate during the data collection period. The study achieved a 100% response rate, as all distributed questionnaires were completed and returned. Eligibility criteria included nurses who had worked in their current hemodialysis unit for at least six months, in order to ensure that participants had sufficient experience and familiarity with the unit’s patient safety practices, workflow processes, and organisational environment. Participants who did not fit the inclusion criteria were excluded.

Data collection instrument

Data were collected using a structured, self-administered questionnaire consisting of two sections. The first section captured sociodemographic and work-related characteristics, including age, gender, marital status, educational qualification, nationality, years of experience, working hours per week, hospital affiliation, and unit of care.

The second section utilised the Hospital Survey on Patient Safety Culture (HSOPSC), a widely validated instrument developed by the Agency for Healthcare Research and Quality to assess healthcare professionals’ perceptions of patient safety culture [10]. The HSOPSC comprises 42 items organised into 12 dimensions, including teamwork within units, supervisor/manager expectations and actions promoting safety, organisational learning and continuous improvement, communication openness, feedback and communication about error, non-punitive response to error, staffing, management support for patient safety, teamwork across units, handoffs and transitions, overall perceptions of patient safety, and frequency of events reported [9,10]. In addition to the 12 dimensions, the instrument includes two outcome variables: the overall patient safety grade and the frequency of events reported within the past 12 months. The patient safety grade provides an overall assessment of safety within the unit, while the frequency of reported events reflects the extent of incident-reporting practices and openness within the organisation [10]. The Hospital Survey on Patient Safety Culture (HSOPSC) is a publicly available instrument developed by the Agency for Healthcare Research and Quality (AHRQ). Permission for its use was obtained in accordance with AHRQ guidelines, and the instrument was used without modification to maintain its validity and reliability. Scores for each dimension are calculated based on the average percentage of positive answers to the items that comprise it. Higher scores reflect a more positive perception of patient safety culture. For interpretation purposes, mean scores of 1.00-2.99 were classified as poor patient safety culture, scores of 3.00-3.99 were classified as moderate patient safety culture, and scores of 4.00-5.00 were classified as good patient safety culture.

Data collection procedure

Data were collected through self-administered questionnaires distributed to eligible participants in the selected hospitals. Participants were informed about the study objectives and procedures and provided informed consent prior to participation. Measures were taken to ensure anonymity and confidentiality, including the use of non-identifiable questionnaires and secure data handling procedures.

Statistical analysis

Data analysis was performed using appropriate statistical techniques on SPSS software (IBM Corp., Armonk, NY). Descriptive statistics, including frequencies, percentages, means, and standard deviations, were used to summarise participant characteristics and HSOPSC dimension scores.

Inferential analysis was conducted using multivariable logistic regression to identify predictors of overall patient safety culture. Logistic regression is commonly applied in cross-sectional studies to assess associations between independent variables and categorical outcomes. Independent variables included demographic characteristics (age, gender, marital status, educational level, and nationality) and work-related factors (hospital, unit of care, years of experience, working hours per week, and number of reported events). Odds ratios (ORs) with 95% confidence intervals (CIs) were reported, and statistical significance was set at p < 0.05.

## Results

Participant characteristics

A total of 400 nurses participated in the study. The majority of respondents were female (n = 269, 67.3%), and the largest age group was 26-35 years (n = 164, 41.0%). More than half of the participants were married (n = 209, 52.3%), and the majority held a Bachelor of Science in Nursing (n = 222, 55.5%). In terms of nationality, 59.5% (n = 238) were Saudi nationals.

Regarding workplace distribution, the highest proportion of participants worked at King Abdulaziz Hospital (n = 129, 32.3%), followed by Al Thaghr Hospital (n = 110, 27.5%) and East Jeddah General Hospital (n = 97, 24.3%). With respect to professional experience, 31.5% (n = 126) of respondents had 3-5 years of experience in their current position, while 35.0% (n = 140) had more than five years of experience in the hospital. Most participants worked less than 50 hours per week (n = 288, 72.0%), and the majority were staff nurses (n = 333, 83.3%). Concerning unit of care, the largest proportion worked in inpatient units (n = 137, 34.3%), followed by surgical units (n = 81, 20.3%) and emergency units (n = 63, 15.8%) (Table 1).

**Table 1:** Demographics and Work-Related Characteristics of Respondents (N = 400)

Variable	Category	Number	%
Gender	Female	269	67.3
	Male	131	32.8
Age	18-25 years	82	20.5
	26-35 years	164	41.0
	36-45 years	106	26.5
	>45 years	48	12.0
Marital status	Single	145	36.3
	Married	209	52.3
	Divorced	27	6.8
	Widow	19	4.8
Educational qualification	Diploma	112	28.0
	BSc nursing	222	55.5
	Postgraduate degree	66	16.5
Nationality	Saudi	238	59.5
	Non-Saudi	162	40.5
Hospital	Adams Hospital	64	16.0
	Al Thaghr Hospital	110	27.5
	East Jeddah General Hospital	97	24.3
	King Abdulaziz Hospital	129	32.3
Work experience (current position)	0-2 years	116	29.0
	3-5 years	126	31.5
	6-10 years	86	21.5
	>10 years	72	18.0
Hours worked per week	<50 hours	288	72.0
	≥50 hours	112	28.0
Years in hospital	0-2 years	133	33.3
	3-5 years	127	31.8
	>5 years	140	35.0
Nursing position	Staff nurse	333	83.3
	Head nurse	67	16.8
Unit of care	Emergency	63	15.8
	General outpatient	47	11.8
	Inpatient unit	137	34.3
	Paediatric	47	11.8
	Psychiatry	25	6.3
	Surgery	81	20.3

Patient safety culture dimensions (HSOPSC)

The overall mean score for patient safety culture was 3.29 (±0.74), indicating a moderate level of positive perception among respondents. Among the 12 dimensions of the HSOPSC, management support for patient safety had the highest mean score (3.37 ± 0.87), followed closely by organisational learning and continuous improvement (3.36 ± 0.88). Other dimensions, including communication openness (3.31 ± 0.75), staffing (3.30 ± 0.77), and handoffs and transitions (3.30 ± 0.72), demonstrated similar moderate scores. Teamwork within units (3.27 ± 0.69), feedback and communication about error (3.27 ± 0.71), and overall perceptions of patient safety (3.28 ± 0.68) also reflected moderate levels. The frequency of events reported dimension recorded the lowest mean score (3.20 ± 0.55) (Table 2).

**Table 2:** Level of the HSOPSC by Dimension The reliability of the HSOPSC questionnaire was high, with a Cronbach’s alpha coefficient of 0.971. SD: standard deviation, HSOPSC: Hospital Survey on Patient Safety Culture

Number	HSOPSC dimensions	Mean	SD
1	Supervisor/manager expectations and actions promoting patient safety	3.28	0.78
2	Organizational learning - continuous improvement	3.36	0.88
3	Teamwork within units	3.27	0.69
4	Communication openness	3.31	0.75
5	Feedback and communication about error	3.27	0.71
6	Non-punitive response to error	3.29	0.70
7	Staffing	3.30	0.77
8	Management support for patient safety	3.37	0.87
9	Teamwork across hospital units	3.28	0.69
10	Handoffs and transitions	3.3	0.72
11	Overall perceptions of patient safety	3.28	0.68
12	Frequency of events reported	3.20	0.55
Overall	HSOPSC questionnaire	3.29	0.74

Overall patient safety grade and event reporting

Regarding overall patient safety grades, most respondents rated patient safety as “very good” (n = 150, 37.5%) or “excellent” (n = 79, 19.8%), while 31.7% (n = 127) rated it as “acceptable” and 11.0% (n = 44) as “poor.” In terms of event reporting, a total of 38.3% (n = 153) reported no events in the past 12 months, while 24.8% (n = 99) reported one to two events. Smaller proportions reported higher frequencies of events, with only 2.0% (n = 8) indicating that they had reported 21 or more events. These findings suggest generally low levels of reported safety incidents among participants (Table 3).

**Table 3:** Overall Patient Safety Grade and Frequency of Reported Events (N = 400)

Variable	Category	Number	%
Overall patient safety grade	Excellent	79	19.8
	Very good	150	37.5
	Acceptable	127	31.7
	Poor	44	11.0
Frequency of reported safety events	No events	153	38.25
	1-2 events	99	24.75
	3-5 events	62	15.5
	6-10 events	52	13.0
	11-20 events	26	6.5
	≥21 events	8	2.0

Predictors of patient safety culture

Multivariable logistic regression analysis identified several significant predictors of overall patient safety culture. Nurses with 3-5 years of experience in their current position were significantly more likely to report higher patient safety culture compared to those with more than 10 years of experience (OR = 2.32, 95% CI: 1.33-4.04, p = 0.003). Working hours were also a significant predictor, as respondents who worked less than 50 hours per week had higher odds of reporting positive patient safety culture compared to those working longer hours (OR = 1.64, 95% CI: 1.10-2.44, p = 0.016).

Event reporting patterns were significantly associated with patient safety culture. Respondents who reported no events (OR = 4.57, p = 0.044), 6-10 events (OR = 5.21, p = 0.034), and 11-20 events (OR = 5.64, p = 0.037) had significantly higher odds of reporting better patient safety culture compared to those reporting more than 21 events. Institutional differences were also observed. No statistically significant associations were found between patient safety culture and gender, age, marital status, educational qualification, nationality, nursing position, or unit of care (Table 4).

**Table 4:** Predictors of Overall HSOPSC Predictors with statistically significant p-values (p < 0.05) are highlighted in bold. OR: odds ratio, CI: confidence interval, HSOPSC: Hospital Survey on Patient Safety Culture

Variable	Category	OR	95% CI (lower-upper)	Wald χ²	df	p-value
Work experience (current position)	0-2 years	1.39	0.79-2.44	1.278	1	0.258
	3-5 years	2.32	1.33-4.04	8.731	1	0.003
	6-10 years	1.47	0.81-2.66	1.596	1	0.206
	>10 years	1.00 (reference)	-	-	-	-
Unit of care	Emergency	0.75	0.40-1.40	0.809	1	0.368
	General outpatient	1.02	0.52-2.01	0.004	1	0.948
	Inpatient unit	1.04	0.63-1.72	0.027	1	0.870
	Paediatric	0.67	0.34-1.32	1.342	1	0.247
	Psychiatry	1.54	0.68-3.50	1.083	1	0.298
	Surgery	1.00 (reference)	-	-	-	-
Nursing position	Staff nurse	0.85	0.52-1.40	0.390	1	0.532
	Head nurse	1.00 (reference)	-	-	-	-
Hours worked per week	<50 hours	1.64	1.10-2.44	5.830	1	0.016
	≥50 hours	1.00 (reference)	-	-	-	-
Years in hospital	0-2 years	1.02	0.67-1.58	0.012	1	0.913
	3-5 years	1.37	0.89-2.10	2.003	1	0.157
	>5 years	1.00 (reference)	-	-	-	-
Event reports (last 12 months)	No events	4.57	1.04-20.05	4.057	1	0.044
	1-2 events	2.91	0.66-12.91	1.977	1	0.160
	3-5 events	3.83	0.84-17.55	2.994	1	0.084
	6-10 events	5.21	1.13-23.98	4.482	1	0.034
	11-20 events	5.64	1.11-28.56	4.369	1	0.037
	≥21 events	1.00 (reference)	-	-	-	-
Gender	Female	0.91	0.62-1.35	0.217	1	0.641
	Male	1.00 (reference)	-	-	-	-
Age	18-25 years	1.18	0.61-2.31	0.246	1	0.620
	26-35 years	0.88	0.49-1.59	0.170	1	0.680
	36-45 years	1.24	0.65-2.33	0.424	1	0.515
	>45 years	1.00 (reference)	-	-	-	-
Marital status	Single	0.44	0.18-1.08	3.212	1	0.073
	Married	0.49	0.21-1.15	2.694	1	0.101
	Divorced	0.66	0.22-2.02	0.529	1	0.467
	Widow	1.00 (reference)	-	-	-	-
Educational qualification	Diploma	1.09	0.62-1.91	0.088	1	0.766
	BSc nursing	1.08	0.66-1.78	0.098	1	0.754
	Postgraduate degree	1.00 (reference)	-	-	-	-
Nationality	Saudi	1.05	0.71-1.54	0.061	1	0.805
	Non-Saudi	1.00 (reference)	-	-	-	-

## Discussion

This multicentre cross-sectional study assessed patient safety culture among hemodialysis nurses in Jeddah, Saudi Arabia, and identified key work-related predictors influencing safety perceptions. The findings demonstrate a moderate overall patient safety culture (mean = 3.29), indicating that while positive safety practices exist, substantial gaps remain, particularly in event reporting and workload-related factors. These results align with previous studies conducted in Saudi Arabia and similar healthcare settings, which have consistently reported moderate safety culture levels among nursing staff [9,10].

The highest-rated dimensions in this study were management support for patient safety and organisational learning/continuous improvement, suggesting that institutional commitment to safety and ongoing quality improvement initiatives are relatively well established. This finding is consistent with earlier research indicating that leadership engagement and organisational learning are critical drivers of patient safety culture, as they foster accountability, encourage reporting, and support continuous system improvement [4,10]. Strong managerial support has been shown to enhance staff confidence in safety systems and promote adherence to established protocols, particularly in high-risk environments such as hemodialysis units.

Despite these strengths, the frequency of events reported dimension recorded the lowest score, highlighting a persistent challenge in incident reporting. This finding suggests the presence of underreporting, which may be attributed to fear of blame, lack of feedback, or perceived ineffectiveness of reporting systems. Similar patterns have been reported in previous studies, where a non-punitive culture was identified as a key determinant of reporting behaviour [9,20]. Underreporting of adverse events limits organisational learning and impedes the identification of system vulnerabilities, thereby compromising patient safety outcomes. In high-risk settings such as hemodialysis, where errors can have immediate and severe consequences, strengthening reporting systems is essential for proactive risk management [5].

The study also revealed that most participants rated patient safety as “very good” or “excellent,” despite reporting low numbers of safety events. This apparent discrepancy may reflect a normalisation of risk or a lack of awareness regarding reportable incidents. Previous research has highlighted that healthcare professionals may perceive safety positively even in the presence of unreported errors, particularly in environments where reporting is not actively encouraged [9]. This finding underscores the need for improved education and awareness regarding incident reporting and its role in enhancing patient safety.

With respect to work-related predictors, nurses with 3-5 years of experience demonstrated significantly higher perceptions of patient safety culture compared to those with longer experience. This may be explained by the balance between clinical competence and engagement, as mid-career nurses often possess sufficient experience while remaining adaptable to organisational policies and safety initiatives. Similar findings have been reported in previous studies, suggesting that experience influences safety attitudes, although the relationship is not always linear [10].

Working hours emerged as a significant predictor, with nurses working less than 50 hours per week reporting better safety culture perceptions. This finding aligns with existing evidence linking excessive workload and fatigue to increased risk of errors and reduced adherence to safety protocols [21]. In hemodialysis settings, where continuous monitoring and precision are required, prolonged working hours may compromise vigilance and decision-making, thereby negatively affecting patient safety. These findings highlight the importance of optimising staffing levels and workload distribution to support safe clinical practice.

Interestingly, event reporting patterns were significantly associated with patient safety culture, with both low and moderate levels of reported events linked to higher safety perceptions. This may indicate that environments encouraging reporting, without punitive consequences, are more likely to foster positive safety cultures. Conversely, extremely high levels of reported events may reflect underlying systemic issues or increased exposure to adverse events. This finding supports the notion that reporting behaviour is both an outcome and a determinant of safety culture, reinforcing the need for balanced and supportive reporting systems [11].

From a theoretical perspective, these findings can be interpreted through the lens of systems-based safety models, which emphasise that patient safety outcomes are influenced by interactions between human factors, organisational processes, and environmental conditions [3]. The moderate safety culture observed in this study suggests that while some protective barriers are in place, gaps remain within the system that may allow errors to occur. Addressing these gaps requires a comprehensive approach that integrates leadership support, effective communication, workload management, and continuous learning mechanisms.

The findings of this study have important implications for clinical practice and healthcare management. First, there is a need to strengthen non-punitive reporting systems to encourage transparency and learning from errors. Second, workload optimisation and staffing adjustments are essential to reduce fatigue-related risks and enhance patient safety. Third, ongoing training and awareness programs should be implemented to improve understanding of patient safety principles and reporting practices. Finally, healthcare institutions should adopt standardised safety interventions, such as checklists and protocols, to reduce variability in care and improve outcomes [13,14].

Strengths and limitations

This study has several strengths, including its multicentre design and relatively large sample size, which enhance the generalizability of the findings within the Jeddah context. Additionally, the use of a validated instrument (HSOPSC) ensures reliability and comparability with other studies. However, certain limitations should be acknowledged. The use of a cross-sectional design limits the ability to infer causality between variables [19]. Furthermore, reliance on self-reported data may introduce response bias, including social desirability bias.

Future research directions

Future studies should consider longitudinal designs to assess changes in patient safety culture over time and evaluate the impact of targeted interventions. Additionally, qualitative approaches may provide deeper insights into the barriers and facilitators of safety culture in hemodialysis settings. Further research exploring the integration of digital tools and real-time monitoring systems may also contribute to enhancing patient safety outcomes in high-risk clinical environments.

## Conclusions

This study assessed patient safety culture among hemodialysis nurses in Jeddah, Saudi Arabia, and identified key work-related predictors influencing safety perceptions. The findings indicate a moderate level of patient safety culture, reflecting the presence of positive safety practices alongside critical gaps, particularly in event reporting and workload-related factors. While dimensions such as management support and organizational learning demonstrated relatively strong performance, the low frequency of reported events highlights persistent challenges in fostering a transparent and non-punitive reporting environment.

Work-related factors, including years of experience and working hours, were found to significantly influence patient safety culture. Nurses with moderate experience and those working fewer hours reported more positive safety perceptions, underscoring the importance of workforce management in promoting safe clinical environments. These findings reinforce the need for healthcare systems to adopt a comprehensive, systems-based approach to patient safety that integrates organizational support, effective communication, and optimal staffing conditions.

## References

[1] Lawati MH, Dennis S, Short SD, Abdulhadi NN (2018). Patient safety and safety culture in primary health care: a systematic review. BMC Fam Pract.

[2] San Jose-Saras D, Valencia-Martín JL, Vicente-Guijarro J, Moreno-Nunez P, Pardo-Hernández A, Aranaz-Andres JM (2022). Adverse events: an expensive and avoidable hospital problem. Ann Med.

[3] Sameera V, Bindra A, Rath GP (2021). Human errors and their prevention in healthcare. J Anaesthesiol Clin Pharmacol.

[4] Braithwaite J, Churruca K, Ellis LA (2024). Resilient health care performance in the real world: fixing problems that never happened. BMC Health Serv Res.

[5] Bano T, Haq N, Nasim A (2023). Evaluation of medication errors in patients with kidney diseases in Quetta, Pakistan. PLoS One.

[6] Pittard JD (2023). Safety monitors in hemodialysis. Handbook of Dialysis Therapy.

[7] Ngema SA, Bale TL, Ramukumba TS (2025). Impact of infection prevention and control quality improvements in haemodialysis facilities: a scoping review. BMC Nephrol.

[8] Albreiki S, Alqaryuti A, Alameri T, Aljneibi A, Simsekler MC, Anwar S, Lentine KL (2023). A systematic literature review of safety culture in hemodialysis settings. J Multidiscip Healthc.

[9] Alrasheeday AM, Alkubati SA, Alqalah TA (2024). Nurses' perceptions of patient safety culture and adverse events in Hail City, Saudi Arabia: a cross-sectional approach to improving healthcare safety. BMJ Open.

[10] Algethami F, Alasmari AS, Alessa MK (2024). Patient safety culture in a tertiary care hospital in Makkah, Saudi Arabia, a cross-sectional study. BMC Health Serv Res.

[11] Chance EA, Florence D, Sardi Abdoul I (2024). The effectiveness of checklists and error reporting systems in enhancing patient safety and reducing medical errors in hospital settings: a narrative review. Int J Nurs Sci.

[12] Gong J, Ma Y, An Y, Yuan Q, Li Y, Hu J (2021). The surgical safety checklist: a quantitative study on attitudes and barriers among gynecological surgery teams. BMC Health Serv Res.

[13] Gul F, Nazir M, Abbas K (2022). Surgical safety checklist compliance: the clinical audit. Ann Med Surg (Lond).

[14] Abd-Elghany S, Abd-Elrahman ME, Nabeh TN, & Rady SH (2023). Efficacy of applying a structured checklist on safety outcomes of hemodialysis patients. Egypt J Health Care.

[15] Marcelli D, Matos A, Sousa F (2015). Implementation of a quality and safety checklist for haemodialysis sessions. Clin Kidney J.

[16] Dhule M, Jacob M (2020). Effect of haemodialysis safety checklist on arterio-venous fistula outcomes among haemodialysis patients. Int Res J Modern Eng Technol Sci.

[17] Alghamdi A, Alaryni A, AlMatham K (2023). Knowledge, attitudes, and practices of high-risk patients towards prevention and early detection of chronic kidney disease (CKD) in Saudi Arabia. Int J Environ Res Public Health.

[18] Mousa D, Alharbi A, Helal I (2021). Prevalence and associated factors of chronic kidney disease among relatives of hemodialysis patients in Saudi Arabia. Kidney Int Rep.

[19] Hess DR (2023). Observational studies. Respir Care.

[20] Lawton R, McEachan RR, Giles SJ, Sirriyeh R, Watt IS, Wright J (2012). Development of an evidence-based framework of factors contributing to patient safety incidents in hospital settings: a systematic review. BMJ Qual Saf.

[21] McHugh MD, Rochman MF, Sloane DM (2016). Better nurse staffing and nurse work environments associated with increased survival of in-hospital cardiac arrest patients. Med Care.

